# Post-transplant lymphoproliferative disorder risk and outcomes in renal transplant patients treated with belatacept immunosuppression

**DOI:** 10.3389/frtra.2023.1280993

**Published:** 2024-01-11

**Authors:** Jean L. Koff, Geeta M. Karadkhele, Jeffrey M. Switchenko, Manali Rupji, Kendra Little, Christian P. Larsen

**Affiliations:** ^1^ Department of Hematology and Medical Oncology, Winship Cancer Institute of Emory University, Atlanta, GA, United States; ^2^ Department of Surgery, Emory University, Atlanta, GA, United States; ^3^ Biostatistics Shared Resource, Winship Cancer Institute of Emory University, Atlanta, GA, United States; ^4^ Office of Information Technology, Emory University, Atlanta, GA, United States

**Keywords:** post-transplant lymphoproliferative disorder (PTLD), belatacept, renal transplant, immunosuppression complication, survival and prognosis, Epstein–Barr virus (EBV)

## Abstract

**Introduction:**

Post-transplant lymphoproliferative disorder (PTLD) is a rare but life-threatening malignancy that arises in the setting of immunosuppression (IS) after solid organ transplant. IS regimens containing belatacept have been associated with an increased risk of PTLD in Epstein–Barr virus (EBV)-seronegative renal transplant recipients, and the use of belatacept is contraindicated in this population. However, the impact of belatacept-based regimens on PTLD risk and outcomes in EBV-seropositive renal transplant recipients is less well characterized.

**Methods:**

A case-control study was conducted to investigate how combinatorial IS regimens impact the risk of PTLD and survival outcomes in renal transplant recipients at a large transplant center between 2010 and 2019. In total, 17 cases of PTLD were identified and matched 1:2 to controls without PTLD by age, sex, and transplanted organ(s). We compared baseline clinical characteristics, examined changes in IS regimen, viral loads, and renal function over time, and evaluated time-to-event analyses, including graft rejection and survival.

**Results:**

Cases of PTLD largely resembled matched controls in terms of baseline characteristics, although expected differences in EBV serostatus trended toward significance (42.9% of PTLD cases were donor-positive/recipient-negative vs. 8.3% controls, *p* = 0.063). PTLD cases were not more likely to have received belatacept than controls. Belatacept was not associated with graft rejection or failure, re-transplant, hospitalization, or decreased survival.

**Conclusions:**

Belatacept was not associated with an increased risk of PTLD, and was not associated with decreased survival in either PTLD cases or in the entire cohort. Our case-control study supports the concept that belatacept remains a safe and effective option for IS in EBV-seropositive renal transplant patients.

## 1 Introduction

Post-transplant lymphoproliferative disorder (PTLD) represents a diverse group of lymphoid diseases that arise in the setting of immunosuppression (IS) after organ transplant. Reported cumulative incidences of PTLD are in the range of 0.6%–2.5% in adult renal transplant recipients (1–3), compared to incidence rates of up to 20% in other transplanted organs (4, 5), which likely reflect differences in the intensity of immunosuppressive regimens used for each type of transplant. Indeed, the primary factors known to impact the risk of PTLD include the extent of T-cell suppression and the Epstein–Barr virus (EBV) serologic status of the recipient. PTLD is grouped into four main histologic categories with divergent clinical prognoses (6): early/non-destructive; polymorphic; monomorphic, which includes aggressive B- and T-cell lymphomas; and classical Hodgkin lymphoma (7). As 50%–70% of PTLDs are EBV-related, PTLD pathogenesis is largely thought to derive from malignant transformation of EBV-infected B cells against the background of the reduced T-cell control that results from IS (5, 8–10). For those cases of PTLD in which EBV's role is not readily apparent, the pathogenic mechanism is less well understood. Importantly, estimates of PTLD-related mortality are in the range of 30%–60% (11).

Belatacept is a fusion protein comprising the Fc fragment of human IgG1 linked to the extracellular domain of cytotoxic T-lymphocyte–associated antigen 4 (CTLA-4) that selectively inhibits T-cell activation through co-stimulation blockade (12) and was approved in 2011 by the U.S. Food and Drug Administration for organ rejection prophylaxis in renal transplant patients (13). Given an early signal for increased risk of PTLD (largely monomorphic subtype involving the central nervous system (CNS)) in EBV-seronegative recipients (14–16), its indication is limited to a less-intensive regimen in EBV-seropositive patients.

In addition to impacting the risk of PTLD development, the IS regimen may also play a role in lymphoma-related survival in certain histologic subtypes (6). The impact of belatacept-based regimens on PTLD risk and survival outcomes in EBV-seropositive renal transplant recipients is not well characterized. The relative rarity of PTLD has made evaluating clinical outcomes in specific patient populations difficult, and few studies have examined PTLD subtypes: larger epidemiologic cohorts often lack granular data on IS regimens, and transplant-based registries may incompletely capture PTLD details, such as histologic subtype. To investigate how combinatorial IS regimens impact the risk of PTLD and survival outcomes in renal transplant recipients in the belatacept era, we conducted a case-control study at a large transplant center with a cohort selected from patients transplanted after 2010.

## 2 Methods

### 2.1 Study cohort

Patients who received a kidney transplant, including kidney-pancreas transplants, between 2010 and 2019 were identified using the Transplant Data Mart (TDM). The TDM is a consolidated transplant data repository that integrates Clinical Data Warehouse, pre-transplant tracking through the Organ Transplant Tracking Record, Nautilus laboratory information systems for lab samples, histocompatibility leukocyte antigen (HLA) matching data from HistoTrac, United Network for Organ Sharing (UNOS) donor data, and REDCap systems into a HIPAA-compliant Oracle database in near real time (<24 h time lag). Information regarding demographics, transplant characteristics, medications, biopsies, and viral studies was pulled from the TDM. The study was approved by our institutional review board (IRB00107934).

### 2.2 Case identification

Cases of PTLD were identified using the presence of ICD-9 and ICD-10 codes in the medical record after the patient received a renal transplant. ICD-10 codes include C81, C83.3, C83.5, C83.7, C83.8, C83.9, C84, C85, C86, C88.8, C88.9, C90, and D47.Z1 and ICD-9 codes include 200.2, 200.5, 200.6, 200.7, 201, 202.0+, 202.1, 202.2, 202.7, 202.8, 202.9, 203, and 238.77 (see the Supplementary Tables). A review of medical records was performed to validate all cases of PTLD identified from the diagnosis codes. To maximize the number of PTLD cases, the institutional pathology archive was queried to identify PTLD cases diagnosed between 2010 and 2019 and cross-referenced with patients within the TDM.

### 2.3 Control matching

Controls were identified from the pool of 2,949 patients (minus the PTLD cases) who underwent renal transplant at Emory between 2010 and 2019, and matched to the case population on age at transplant with buffer ±5 years, sex, transplanted organ (i.e., kidney vs. kidney-pancreas), and race. The match was conducted using a Python script from the Berkeley Source Distribution (BSD)-licensed Python Data Analysis Library (Pandas) (17) that optimized matches to ensure the highest number of control matches for each given case within the match criteria.

### 2.4 Statistical analyses

Descriptive statistics were generated for all patient characteristics, including IS regimen, sex, race, ethnicity, donor type, and age at transplant. The IS regimen was determined using pharmacy order data in the electronic medical record that specified “initial protocol” and validated using the medical administration record in the first week after the transplant. Frequency and percentage were reported for categorical variables, and median and interquartile range (IQR) were reported for numeric variables. Differences between cases and controls for each clinicopathological variable were assessed using conditional logistic regression to account for matching.

To specifically evaluate outcomes related to the IS regimen, only cases and controls with complete data on IS regimen were included in survival analyses. Overall survival (OS) was estimated using the Kaplan–Meier method. For comparison between IS groups within the PTLD cases, 10 cases with available IS and survival data were identified, with OS compared from date of PTLD diagnosis. To compare OS between PTLD cases and controls, the subset cohort with complete data on IS regimen and preserved 1:2 case-control matching included 7 PTLD cases and 14 matched controls. In this subset cohort (*n* = 21), OS was calculated from date of transplant and compared using log-rank tests.

A univariate Cox regression analysis was used to determine the effect of each clinicopathological variable on OS. To test the effect of treatment on the other secondary outcomes (i.e., graft failure, re-transplant, graft rejection, hospitalization, and number of rejection episodes), conditional logistic regression or exact conditional Poisson or linear regression was performed, as appropriate. Statistical analysis was performed using SAS 9.4 (SAS Institute Inc., Cary, NC, USA), and statistical significance was assessed at the 0.05 level.

### 2.5 Clinical event mapping

To assess how changes in IS, graft function, and viral loads (of cytomegalovirus (CMV), EBV, and BK virus) correlated temporally with discrete events such as PTLD diagnosis, hospitalization, and graft rejection or loss, we generated clinical event maps that captured dynamic changes in these variables over time relative to outcomes of interest. Laboratory values such as creatinine, estimated glomerular filtration rate (e-GFR), and IS agent levels were plotted as continuous variables. Events were captured from date of transplant until the date of last follow-up.

## 3 Results

### 3.1 Cohort characteristics

A total of 17 patients with PTLD were identified, with their histology detailed in Table 1. Excluding the two cases of PTLD who underwent a second renal transplant after PTLD diagnosis, the median time to PTLD diagnosis from transplant was 11.0 months. As detailed in Table 2, cases of PTLD did not differ significantly from controls in terms of baseline variables. Because none of the PTLD cases had undergone kidney-pancreas transplants, only renal transplant patients were included in the case-control cohort to preserve matching on organ transplant type. Differences in EBV serostatus trended toward significance, with 42.9% of PTLD cases being donor-positive/recipient-negative compared to 8.3% of controls (*p* = 0.063); this was expected, as recipient EBV seronegativity is a well-described risk factor for PTLD. In the entire cohort, detailed data regarding the IS regimen were available for 21 patients; the IS regimen did not change from the initial protocol over time. Patients treated with belatacept did not differ from patients treated with tacrolimus or other non-belatacept IS regimens in terms of sex, race, donor type, or age at transplant. Importantly, no EBV-seronegative transplant recipients received belatacept.

**Table 1:** Histology of PTLD cases identified in a single-center cohort of renal transplant recipients, 2010–2019.

Histology	*N* = 17 (%)
Monomorphic	11 (64)
Diffuse large B-cell lymphoma	10 (59)
With CNS involvement	2 (12)
Plasmacytoid	1 (6)
Classical Hodgkin	1 (6)
Polymorphic	2 (12)
Non-destructive	3 (18)

**Table 2:** Differences between PTLD cases and age-, sex-, race- and transplanted organ-matched controls in a single-center cohort of renal transplant recipients, 2010–2019.

Variable	Cases *n* = 17 (%)	Controls *n* = 34 (%)	Total *n* = 51 (%)	*p*-value
Median follow-up time from transplant, months	103.7	49.8	57.1	N/A
Median age at transplant, years	48.23	47.73	47.8	0.392
Interquartile range	31.8–57.1	32.2–57.9	31.8–57.9	
Sex				N/A
Male	10 (58.8)	20 (58.8)	21 (41.2)	
Female	7 (41.2)	14 (41.2)	30 (58.8)	
Race				N/A
African American	5 (29.4)	10 (29.4)	15 (29.4)	
Non-African American	12 (70.6)	24 (70.6)	36 (70.6)	
Ethnicity				0.624
Hispanic	1 (6.3)	1 (3.2)	2 (4.3)	
Non-Hispanic	15 (93.8)	30 (96.8)	45 (95.7)	
Unknown	1	3	4	
Donor type				0.739
Deceased	10 (58.8)	20 (58.8)	30 (61.2)	
Living	6 (35.3)	13 (38.2)	19 (38.8)	
Unknown	1	1	2	
Re-transplant				0.773
Yes	2 (11.8)	5 (14.7)	7 (13.7)	
No	15 (88.2)	29 (85.3)	44 (86.3)	
EBV serostatus				0.063
Donor−/recipient −	1 (7.1)	0	1 (2.6)	
Donor+/recipient −	6 (42.9)	2 (8.3)	8 (21.1)	
Donor−/recipient +	0	1 (4.2)	1 (2.6)	
Donor−/recipient +	7 (50.0)	21 (87.5)	28 (73.7)	
Unknown	3	10	13	
Immunosuppression				0.438
Belatacept	3 (27.3)	9 (47.4)	12 (40)	
Tacrolimus/other	8 (72.7)	10 (52.6)	18 (60)	
Unknown	6	15	21	
Graft rejection of any grade^a^				0.512
Yes	12 (54.6)	5 (41.7)	17 (50)	
No	10 (45.4)	7 (58.3)	17 (50)	
Unknown	5	12	17	
Graft loss				0.0957
Yes	4 (23.5)	2 (5.9)	6 (11.8)	
No	13 (76.5)	32 (94.1)	45 (88.2)	

*p*-values calculated using exact conditional logistic regression.

^a^ Rejection grades included grades 1, 2, 3, and borderline.

### 3.2 Clinical event mapping

**Figure 1:**
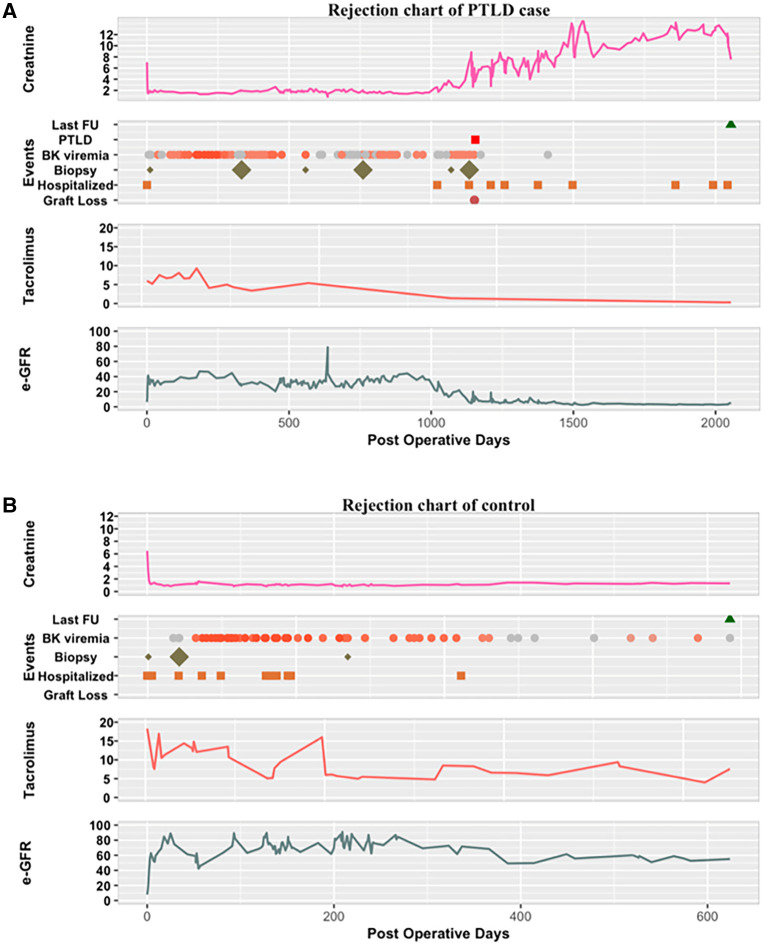
Representative clinical event maps of (**A**) PTLD case and (**B**) control patients from date of transplant until date of last follow-up. For BK viremia plots, gray circles signify undetectable levels, with darkening orange gradient signifying higher viral levels. For biopsy plots, small diamond signifies grade 1 rejection, large diamond signifies higher grade of rejection by pathology. Creatinine, serum creatinine; FU, follow-up; tacrolimus, trough serum levels of tacrolimus.

A comparison of dynamic events in the clinical event maps did not reveal any discernible patterns in changes in IS level, viral load, or PTLD diagnosis relative to biopsy events, graft loss, or hospitalization. Representative maps are shown in Figure 1.

### 3.3 Survival and transplant outcomes

**Figure 2:**
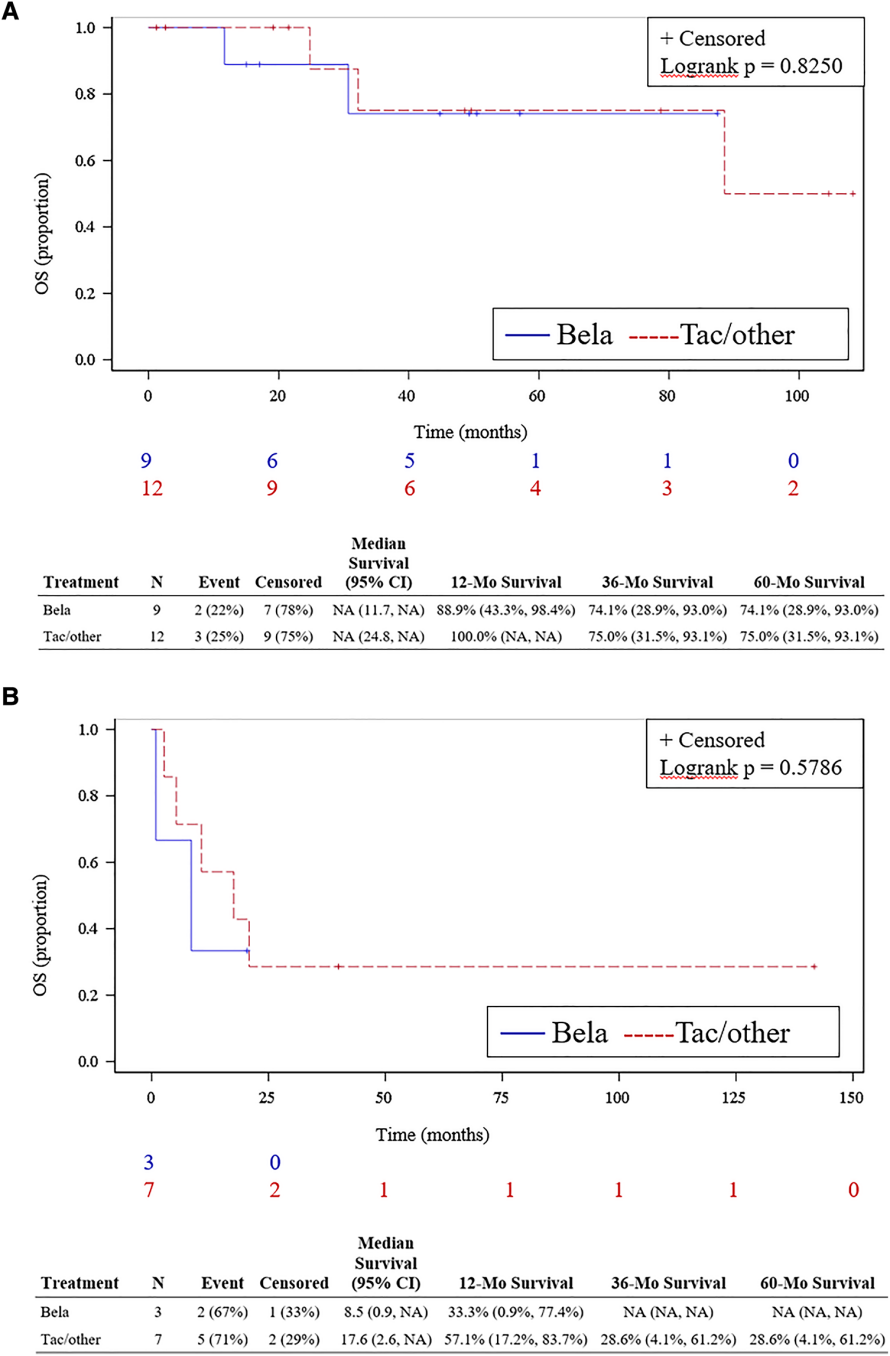
OS by immunosuppression regimen in PTLD case-control cohort. (**A**) OS from date of transplant in PTLD cases (*n* = 7) and matched controls (renal transplant recipients, *n* = 14) from selected cohort of patients with detailed clinical and survival data. (**B**) OS from PTLD diagnosis in PTLD cases with detailed clinical and survival data (*n* = 10). Bela, belatacept; tac, tacrolimus-containing regimens.

For the 21 patients with detailed IS data, belatacept treatment, sex, race, and age at transplant were not associated with OS in univariate analysis; deceased donor transplant trended toward significance compared to living donor transplant, with a hazard ratio of 6.84 (95% confidence interval (CI) 0.72–64.97, *p* = 0.058). IS with belatacept was not associated with an increased risk of graft rejection (either event or number events), graft failure, re-transplant, or hospitalization. Belatacept was not associated with OS in either the selected cohort of matched cases and controls with detailed IS data (*n* = 21, calculated from date of transplant) (Figure 2A) or in the entire cohort of PTLD patients with detailed IS and survival data (*n* = 10, calculated from the date of PTLD diagnosis) (Figure 2B).

## 4 Discussion

Our results serve as an initial “real-world” characterization of PTLD risk and outcomes in the era of belatacept treatment. The matched case-control design allowed for the evaluation of risk factors for and histologic subtypes of PTLD, a relatively rare but life-threatening complication of kidney transplant; this approach was complemented by novel clinical mapping of transplant-related outcomes relative to longitudinal changes in important variables, such as viremia and IS regimens. Although our large institutional database provided a sizable pool from which to identify cases and matched controls, our work was subject to the limitations inherent to retrospective single-institution studies, particularly with regard to missing data elements. For some patients who received transplants at an outside center, pre-transplant information, such as EBV serostatus and donor type, were not available either in our electronic medical records or via institutional queries of UNet, UNOS’ online database system; immunosuppression is not a required data element for UNet. Similarly, post-transplant events such as graft rejection are not adequately captured for patients who transition care to outside centers after transplant at our institution. To minimize the impact of these missing data and outcomes, we performed focused analyses on a subset of cases and controls for whom clinical data were complete. Importantly, we did not detect an increased use of belatacept-containing IS regimens in PTLD cases compared to matched controls; the small sample size precluded a meaningful subgroup analysis limited to EBV-seropositive patients. Our findings are in line with several other recently presented studies of EBV-seropositive belatacept-treated renal transplant recipients. An analysis of individuals in the Organ Procurement and Transplantation Network (OPTN) database transplanted between 2011 and 2016 also described a low risk of PTLD in this group in routine clinical practice (18). Highlighting the rarity of PTLD, and perhaps indicative of some of the difficulty in adequately capturing its diagnosis and outcomes, that study detected only nine cases of PTLD (two with known CNS involvement) from a cohort of 1,631 belatacept-treated patients. Similarly, a study of the long-term safety of belatacept using the prospective, multicenter EnLiST registry detected only four cases of PTLD (one with CNS involvement) from a cohort of 933 EBV-seropositive patients; the cumulative incidence rate was estimated at 0.08 per 100 patient-years for non-CNS-PTLD and 0.03 for CNS-PTLD (19). Post-marketing studies aimed at comparing PTLD incidence rates in belatacept- vs. calcineurin inhibitor-treated EBV-seropositive kidney transplant recipients are ongoing (e.g., NCT01656343).

Multiple groups have demonstrated that lymphoma-specific mortality is higher in patients with PTLD compared to patients with *de novo* lymphoma with a corresponding histologic subtype (20, 21), underscoring the fact that multiple factors may lead to decreased survival in transplant patients with cancer, including complications of end-organ damage and IS-related infections. In the case of PTLD, both IS reduction and chemo-immunotherapy can help to eliminate the malignancy but also increase the risks of treatment-induced toxicity and organ graft rejection. In addition, while it is well established that certain IS agents are associated with increased PTLD risk, little is known about the impact of these agents on outcomes once PTLD develops, especially given the heterogeneity in survival seen among various PTLD subtypes (6). In our cohort, patients with PTLD were not more likely than matched controls to experience graft rejection of any kind, and the receipt of belatacept did not increase risk for graft rejection events. Although our cohort's relatively small sample size limited our power to utilize multivariable survival models, belatacept IS did not appear to increase PTLD-related mortality, which was expectedly high.

Overall, our case-control study supports the concept that belatacept remains a safe and effective option for IS in EBV-seropositive renal transplant patients. Our work adds weight to recently reported safety data from registries of belatacept-treated patients, providing further granularity regarding observed PTLD histologies. In addition, we have developed a tool that helps visualize dynamic changes in key factors impacting transplant and PTLD outcomes. Future studies are needed to investigate the intricate interplay between IS regimens, patient comorbidities, and tumor biology that likely underlies inferior survival in patients with PTLD.

## Data Availability

The data analyzed in this study are subject to the following licenses/restrictions: The dataset is encrypted, de-identified, and compliant with the Health Insurance Portability and Accountability Act (HIPAA). Each patient is assigned a study-specific code, and a distinct key is employed to connect the study-specific code with patient identifiers. Access to the database and its corresponding key is limited exclusively to the study team and the principal investigator. This information is considered confidential and will not be shared with any external parties. For inquiries regarding access to these datasets, kindly direct requests to kbryan9@emory.edu.
