# Supplementary material for: Post-transplant lymphoproliferative disorder risk and outcomes in renal transplant patients treated with belatacept immunosuppression
Source: Front Transplant. 2024 Jan 11;2:1280993. doi: 10.3389/frtra.2023.1280993 (PMC11235367; doi:10.3389/frtra.2023.1280993)
Supplement: Supplementary file 1 [file Datasheet1.docx]

**Supplemental Tables. ICD-9 and ICD-10 codes used to identify post-transplant lymphoproliferative disorder (PTLD) cases.**

| **ICD-9 code** | **Diagnostic entity** |
| --- | --- |
| 200.2 | Burkitt's tumor or lymphoma |
| 200.5 | Primary central nervous system lymphoma |
| 200.6 | Anaplastic large cell lymphoma |
| 200.7 | Large cell lymphoma |
| 201 | Hodgkin's disease |
| 202.0+ | Nodular lymphoma |
| 202.1 | Mycosis fungoides |
| 202.2 | Sezary's disease |
| 202.7 | Peripheral t-cell lymphoma |
| 202.8 | Other malignant lymphomas |
| 202.9 | Other and unspecified malignant neoplasms of lymphoid and histiocytic tissue |
| 203 | Multiple myeloma and immunoproliferative neoplasms |
| 238.77 | Post-transplant lymphoproliferative disorder (PTLD) |

| **ICD-10 code** | **Diagnostic entity** |
| --- | --- |
| C81 | Hodgkin lymphoma |
| C83.3 | Diffuse large B-cell lymphoma |
| C83.5 | Lymphoblastic (diffuse) lymphoma |
| C83.7 | Burkitt lymphoma |
| C83.8 | Other non-follicular lymphoma |
| C.83.9 | Non-follicular (diffuse) lymphoma, unspecified |
| C84 | Mature T/NK-cell lymphomas |
| C85 | Other specified and unspecified types of non-Hodgkin lymphoma |
| C86 | Other specified types of T/NK-cell lymphoma |
| C88.8 | Other malignant immunoproliferative diseases |
| C88.9 | Malignant immunoproliferative disease, unspecified |
| C90 | Multiple myeloma and malignant plasma cell neoplasms |
| D47.Z1 | Post-transplant lymphoproliferative disorder (PTLD) |
